# Less contrast, more clarity, innovative visualization technique for management of multiple colorectal liver metastases using microwave ablation through a portal venous access

**DOI:** 10.1016/j.radcr.2024.01.038

**Published:** 2024-02-03

**Authors:** Kelly Trinh, Muhammad Hamza Shamim, Mohammad Ghasemi-Rad

**Affiliations:** a School of Medicine, Texas Tech University Health Sciences Center, Lubbock, TX, USA; b Baylor College of Medicine, Houston, TX, USA; c Division of Interventional Radiology, Department of Radiology, Baylor College of Medicine, Houston, TX, USA

**Keywords:** Microwave ablation, Portal vein access, Hepatic metastasis

## Abstract

Colorectal cancer, a leading cause of cancer-related deaths, often results in liver metastases, with about half of patients affected. For those ineligibles for surgery, percutaneous microwave ablation (MWA) offers a viable alternative. Conventionally, visualizing liver lesions prior to MWA demands significant IV contrast, often needing repeated sessions. We introduce a technique using minimal IV contrast to treat multiple metastatic lesions simultaneously. A case of a 47-year-old male with stage 4 colorectal adenocarcinoma and multiple liver metastases is presented. Instead of the typical 100-150 cc of IV contrast, our method used just 25 cc, successfully ablating 6 hepatic metastases in 1 session. This approach not only reduces contrast volume but also optimizes treatment efficiency.

## Background

Colorectal cancer is the second leading cause of cancer death worldwide, and approximately 50% of patients with colorectal cancer develop liver metastases [1]. Percutaneous microwave ablation (MWA) has gained popularity as an alternative treatment option for patients with colorectal liver metastases who are not candidates for surgical intervention [2]. Usually, to visualize small liver lesions during a procedure, a contrast-enhanced portal venous phase computed tomography (ceCT) of the abdomen is performed prior to MWA. This requires an administration of approximately 100-150 cc of intravenous (IV) contrast for each session. For patients with numerous metastatic lesions, this process must be repeated multiple times, demanding a substantial amount of contrast or procedure in multiple days and significant time investment. We herein describe an innovative technique of using MWA with a much small amount of IV contrast to treat multiple metastatic lesions in 1 setting with a representative case.

## Case report

A 47-year-old male presented with stage 4 colorectal adenocarcinoma (metastatic to the liver) diagnosed in 2021 status-post multiple chemotherapy regimens, now on maintenance

**Fig. 1:**
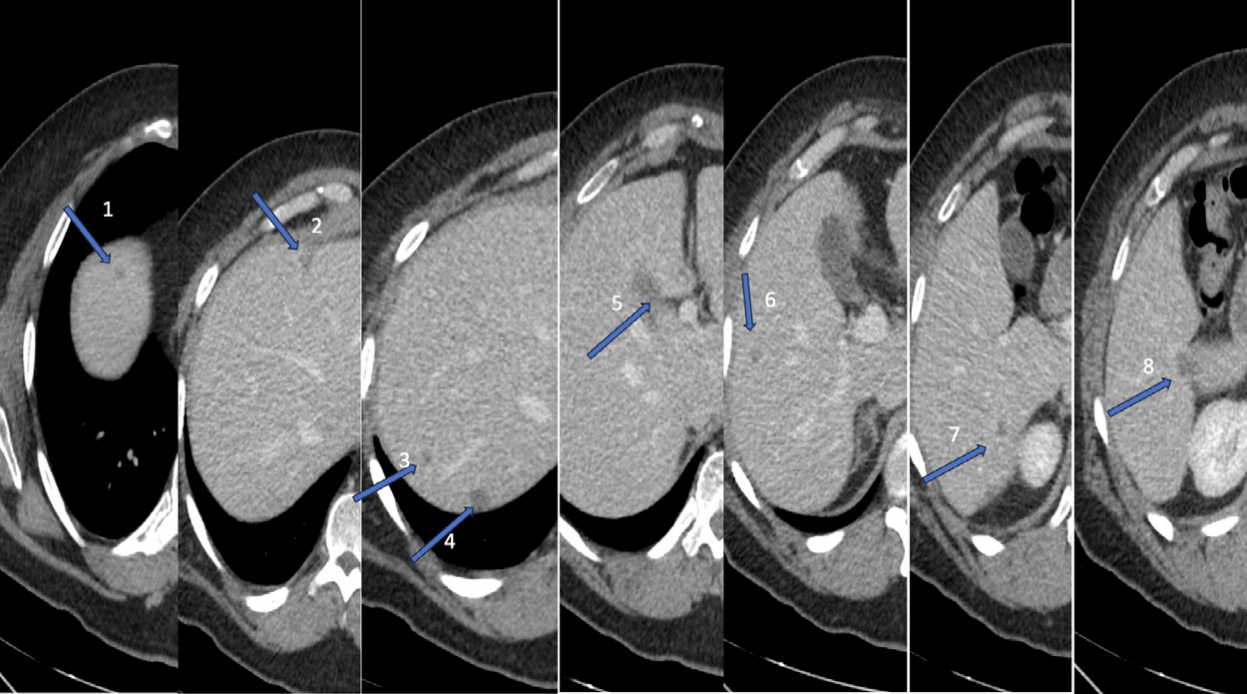
Preprocedural ceCT demonstrates multiple small hepatic lesions consistent with colorectal metastasis (arrows indicates the lesions).

XELODA. Restaging scan showed a mixed response after 1 year of CTX with liver lesions decreasing in size. Per imaging, patient had 8 small lesions (range 0.5–1.5 cm) within the liver, a majority (except one in the segment IV) of which were in the right lobe (Fig. 1). Most were very small and only visualized on portal venous phase of liver CT.

**Fig. 2:**
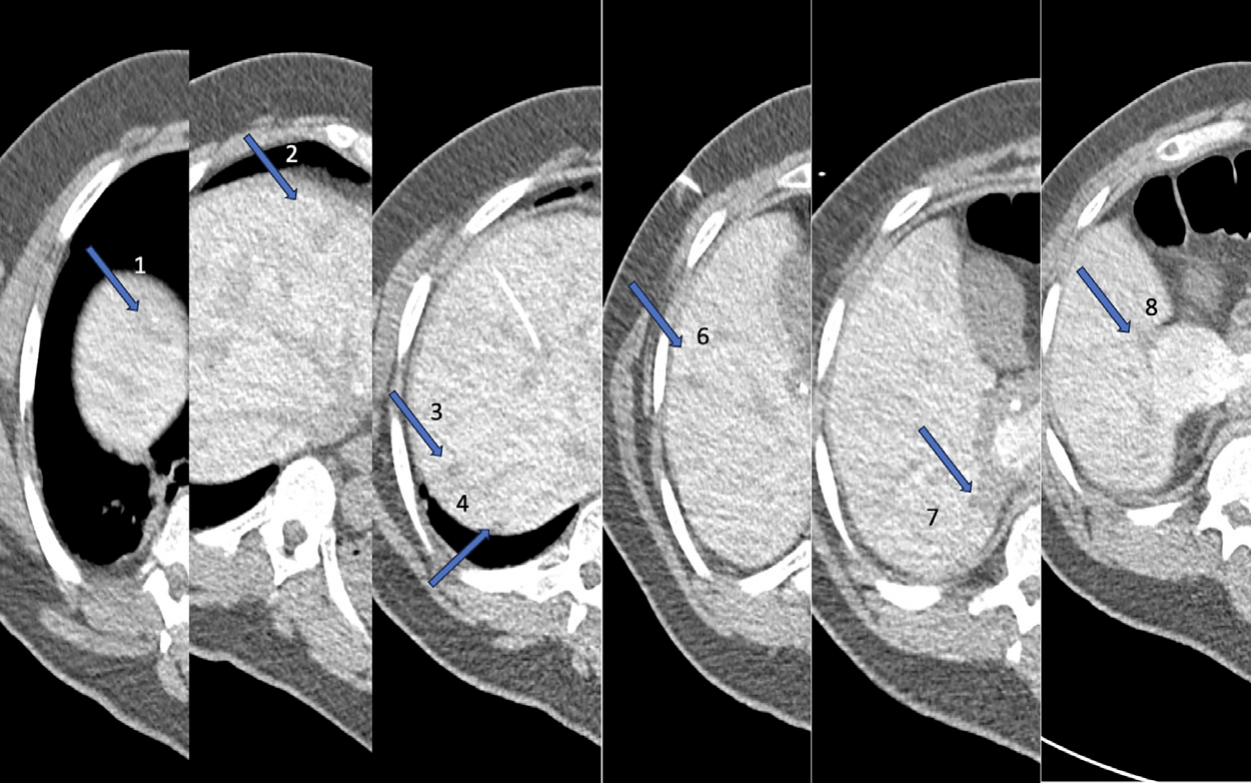
Contrast injection through portal venous catheter demonstrated great visualization of metastatic lesions. Also, partially visualized portal catheter (arrow indicates the lesions).

**Fig. 3:**
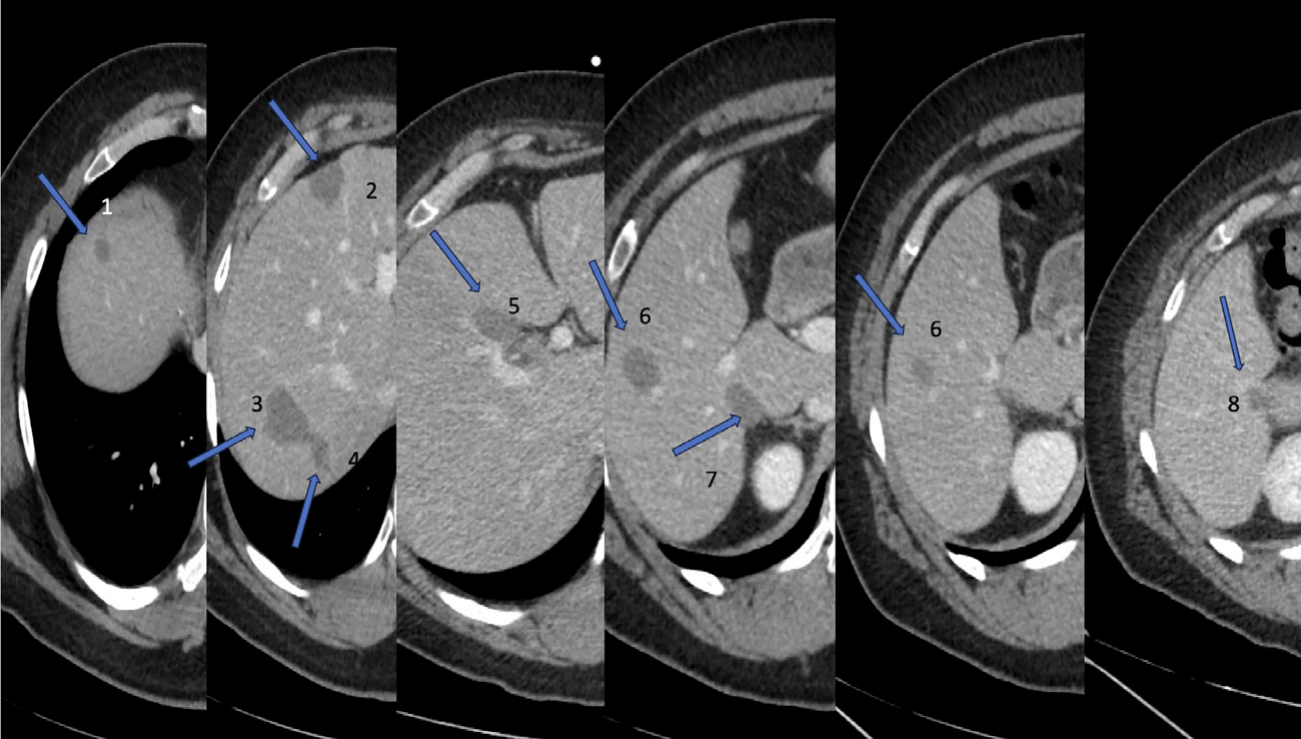
Postprocedural ceCT demonstrates complete multiple ablation cavities consistent with complete treatment.

For the treatment of metastatic lesions in the liver, the patient was given the options of either right hepatectomy with wedge resection/MWA of the segment IV liver lesion or MWA of lesions alone. The patient refused surgery and agreed to proceed with MWA after considering different treatment options. MWA is an excellent alternative modality for treating colorectal liver metastases, and it is often recommended for patients who are not surgical candidates and patients who prefer minimally invasive procedures over liver resection [3]. On the day of the procedure, a CT scan of the abdomen without contrast was performed, which did not reveal most of the lesions. Prior to microwave ablation, transhepatic portal venous access was obtained with US and CT guidance with placement of a 3F microcatheter (Inner dilator of Neff percutaneous access set; cook, Bloomington, Indiana). Before each ablation 5 cc of 25% contrast was injected through the catheter (This was repeated 4 times since 3 lesions were close to each other), and imaging was performed 10-15 seconds after injection for accurate visualization of the lesions. In total, 25 cc of contrast was used (Fig. 2). Using CT guidance, a microwave ablation probe (Solero, Angiodynamic, Latham, New York) was advanced into multiple hepatic masses with successful ablation of the hepatic dome lesion, 3 hepatic segment V/VI lesions, 2 hepatic segment VII lesions (Total 6). All in all, successful CT-guided microwave ablation of 6 hepatic metastases within the right lobe was performed. Notably, the largest metastatic lesion in the gallbladder fossa was not treated due to its close proximity to the gallbladder. After thorough consideration in the tumor board, we decided to proceed with an en-bloc resection of the gallbladder along with lesion . A ceCT performed in the portal venous phase 1 month after the procedure confirmed ablation within the expected region with ablation zones overlapping the known metastatic tumors (Fig. 3). The other lesion (2 lesions, 1 missed, and 1 new since last exam) were ablated on a subsequent day (6 weeks apart), with the previous ablation zone serving as a landmark to guide the correct placement of the MWA antenna. The patient has been under observation for 10 months and has shown no signs of recurrence.

## Discussion

In the conventional approach, approximately 100-150 cc of contrast is administered via a peripheral IV access [4]. The utilization of ceCT facilitates the visualization of each hepatic lesion and the advancement of MWA probe into the target lesion for ablation. This procedure necessitates repetition for each lesion, resulting in a time-consuming process. Furthermore, this approach raises concerns for the patient safety due to the significant quantity of contrast required as well as the multiple anesthesia session. With our new protocol for MWA of multiple metastatic lesions, all known metastases can be identified and ablated within a single session with a significantly lower amount of IV contrast. However, this approach requires a direct transhepatic portal venous access, and 1 major concern is the risk of postprocedural hemorrhage. Bleeding following percutaneous direct portal vein access without transhepatic tract closure has been reported to exceed 30% [2]; however, this only happens in cases where the access is obtained for portal intervention with placement of at least 5 French sheath with outer diameter of 7 French. Regarding our patient, we made efficient use of a small-sized access catheter (3 French in this patient) and the long tract, eliminating the need for tract closure. Furthermore, we employed 2.45 GHz (operating frequency 900-2450 MHz), which is within the typical frequency range for MWA [5]. MWA is also a relatively quick procedure. With microwave energy, a large and consistent zone of ablation can be created in a short period of time [6]. The total duration for our procedure was 1.5 hours, averaging 15 minutes per hepatic lesion. Interestingly, the literature reported greater precision and shorter procedural time, especially with hepatic lesions adjacent to vessels up to 10 mm in size, when using microwave energy compared to radiofrequency [6].

A contrast-enhanced study performed in the portal venous phase at the termination of the procedure and 30 days later confirmed ablation within the expected region with ablation zones overlapping the known metastatic tumors. All in all, successful CT-guided microwave ablation of 6 hepatic metastases within the right lobe was performed.

To our best knowledge, this approach of managing multiple hepatic metastases has not been documented in the literature [7]. This novel approach proves to be a time-efficient solution for both the patient and the interventionalist. Furthermore, only 25 cc of contrast was administered in total, implying the safety and efficacy of this new approach.

## Conclusion

This innovative technique is timesaving for both the patient and the interventionalist since all procedures can be performed in a single session with a considerably low amount of IV contrast. This not only signifies the reliability of this new approach but also eliminates the need for subsequent ablation and anesthesia sessions for treating the remaining lesions as is typical with the conventional approach.

## Patient consent

Informed written consent was obtained from the patient.
